# The Interplay between Age and Viral Status in EBV-Related Nasopharyngeal and HPV-Related Oropharyngeal Carcinoma Patients

**DOI:** 10.3390/cancers14246170

**Published:** 2022-12-14

**Authors:** Stefano Cavalieri, Paolo Bossi, Gabriele Infante, Rosalba Miceli, Nicola Alessandro Iacovelli, Eliana Ivaldi, Laura Deborah Locati, Cristiana Bergamini, Carlo Resteghini, Imperia Nuzzolese, Salvatore Alfieri, Elena Colombo, Rossana Ingargiola, Marzia Franceschini, Giuseppina Calareso, Lisa Licitra, Ester Orlandi

**Affiliations:** 1 Head and Neck Medical Oncology Department, Fondazione IRCCS Istituto Nazionale dei Tumori, 20133 Milan, Italy; 2 Department of Oncology and Hemato-Oncology, University of Milan, 20122 Milan, Italy; 3 SSD Clinical Epidemiology and Trial Organization, Department of Epidemiology and Data Science, Fondazione IRCCS Istituto Nazionale dei Tumori, 20133 Milan, Italy; 4 Department of Clinical Sciences and Community Health & Department of Economics, Management and Quantitative Methods, University of Milan, 20122 Milan, Italy; 5 Radiotherapy Department, Fondazione IRCCS Istituto Nazionale dei Tumori, 20133 Milan, Italy; 6 Radiology Department, Fondazione IRCCS Istituto Nazionale dei Tumori, 20133 Milan, Italy

**Keywords:** elderly, nasopharyngeal carcinoma, oropharyngeal carcinoma, HPV, EBV, prognosis

## Abstract

**Simple Summary:**

We analyzed 324 patients affected by loco-regionally advanced virus- and non-virus-related head and neck cancers treated with curative intent. We aimed at assessing the interplay between age and viral status on outcome (disease-free- and overall survivals) in these patients. We found that old patients had more comorbidities, and received less intensive treatments when compared to younger subjects. OS and DFS were shorter in older patients. However, after adjusting the models for stage, smoking, comorbidities, treatment strategy and dose intensity, no significant differences in terms of survival were observed according to age. Therefore, factors such as comorbidities, treatment intensity and stage have a prognostic role with differential impact on both virus and non-virus related tumors. Age should be considered as the expression of an array of host- and tumor-related features rather than an independent prognostic factor.

**Abstract:**

Background. The aim of this work was to analyze the interplay between age and viral status on the outcomes in loco-regionally advanced oropharyngeal and nasopharyngeal cancer patients treated with radiotherapy and different chemotherapy combinations. Methods. A retrospective (2006–2017) analysis was performed on non-metastatic loco-regionally advanced oropharyngeal (both HPV+ and HPV−) and EBV+ nasopharyngeal cancer patients (young: <65 years vs. elderly: ≥65 years) treated with radiotherapy with or without chemotherapy. The impact of age and viral status on overall (OS) and disease-free survival (DFS) were studied with multivariable models, which were adjusted for smoking, stage, comorbidities, chemotherapy dose intensity and treatment strategy. Results. We analyzed 324 patients (146 HPV+ oropharynx, 63 HPV−, 115 nasopharynx). Elderly patients had more comorbidities, and received less intensive treatments when compared to younger subjects. Although OS and DFS were shorter in older patients, after adjustment for stage, smoking, comorbidities, treatment strategy and dose intensity, no significant differences in terms of survival were observed according to age (65 vs. 50 years of age: HR 1.89, 95% CI 0.45–7.84 for HPV+ OPC; HR 0.91, 95% CI 0.29–2.89 for HPV− OPC; HR 1.99, 95% CI 0.9–4.39 for NPC; *p* = 0.395). Conclusions. Several potential age-related (comorbidities, treatment intensity) and disease-related (stage) confounding factors play a prognostic role with differential impacts on both virus and non-virus-related tumors. In HPV+ oropharyngeal cancer and in EBV+ nasopharyngeal cancer patients, age should be considered as the expression of an array of host- and tumor-related features rather than an independent prognostic factor.

## 1. Introduction

In the last decade, relevant changes in global head and neck cancer (HNC) epidemiology have occurred, essentially driven by the increase of human papillomavirus (HPV)-related carcinomas. Recently, the worldwide prevalence of HPV-associated oropharyngeal cancer (OPC) was reported to be 44.8% [1]; in Europe, a recent meta-analysis showed that the HPV-positive OPC prevalence ranged from 18% to 65% [2].

Virus-related oncogenesis is a common theme in nasopharyngeal cancer (NPC). In regions where NPC is endemic, most cases consist of non-keratinizing subtypes, which are invariably associated with Epstein-Barr virus (EBV) infection. In non-endemic areas, an increase of EBV-related NPC subtypes has been reported across ethnicities and in both genders in the United States [3].

Although literature data report a minority (8%) of HPV positivity in non-keratinizing undifferentiated carcinoma patients in endemic areas, in non-endemic regions this prevalence is unknown and the presence of HPV is more related to keratinizing NPC [4]. Moreover, in clinical practice, HPV is usually not tested in EBER+ NPC, and the same happens for EBV assessment in HPV+ and HPV− OPC patients. 

Although NPC incidence has declined gradually worldwide over the past decades [5], the old-age ratio (total number of individuals aged > 65 years per 100 working age people (20–64 years)) is projected to increase from 13 elderly subjects per 100 in 2010 to 45 per 100 by 2050 [6]. 

Due to the improvement in life expectancy and the influence of preventive HPV vaccination, an escalating incidence in the proportion of HPV-associated OPC among elderly patients has been occurring, and is expected in the next decades [7].

A handful of studies have already reported that the majority of elderly patients with OPC were found to be HPV+, underlying the pivotal role of HPV status in the elderly population [8,9,10].

Since the number of old patients with OPC and NPC will increase with the rapid growth of the elderly population, there is a strong need for data regarding the outcome of this population. 

For HPV-positive OPC very elderly patients, a shift towards non-surgical therapy, possibly due to the known favorable response of HPV-associated tumors to radiotherapy (RT) and/or poor surgical candidacy, has been reported in recent years, without a detrimental impact on survival. However, the best treatment modality has not yet been determined [11].

This is one of the major challenges in oncology because of the need for intense multi-modality regimens in a potentially vulnerable population [12]. 

Given the scarce outcome data for elderly patients with OPC and NPC, we aimed to analyze the interplay between patient age and disease viral status in a retrospective consecutive series of OPC and NPC patients treated with definitive radiotherapy and different combinations of systemic treatments at a single tertiary cancer center. 

## 2. Materials and Methods

### 2.1. Study Population

We conducted a retrospective analysis of consecutive non-metastatic loco-regionally advanced OPC and EBV-positive NPC patients, treated with curative intent at our institution with definitive intensity-modulated radiotherapy (IMRT) techniques with or without systemic treatments (induction and/or concurrent), between 2006 and 2017. Disease was staged according to the eighth edition of the American Joint Committee on Cancer (AJCC) and Union for International Cancer Control (UICC) staging system (AJCC 8th). Inclusion criteria for the study were as follows: pathologically proven OPC with known p16 or HPV status or NPC with known EBV status; stage III-IVa/b (HPV-negative OPC) or stage III-IVa (NPC) or stage I-III (HPV-positive OPC); availability of data about treatment and outcome. Exclusion criteria were: unavailability of HPV and p16 status for OPC patients; unavailability of EBV status for NPC patients; presence of distant metastases; treatments with palliative intent; not completing the curative treatments.

To study the differences between virus-related and unrelated tumors, at a first stage four categories were considered: HPV-positive OPC, EBV-related NPC (virus-related cancers), HPV-negative OPC, EBV-negative NPC (virus-unrelated cancers). HPV status was assessed in OPC patients based on tumor p16 expression (by immunohistochemical analysis) and/or HPV DNA status (by in situ hybridization on tumor specimen). EBV virus-encoded small RNAs (EBER) status was assessed for NPC patients. 

Due to the limited number of EBV-negative NPC, the present analysis included EBER-positive patients only. 

Therefore, three groups were considered in the analysis: two were virus-related (HPV + OPC, EBER + NPC), and one virus-unrelated (HPV− OPC).

The Ethical Committee of our institution approved this study on 19 June 2020 (internal study identifier INT 121-20). Informed consent was obtained from patients, and the study was conducted in accordance with the Declaration of Helsinki.

### 2.2. Treatment 

For both OPC and NPC patients, radiotherapy (RT) was delivered by relying either on a conventional static-field technique (conventional IMRT) or volumetric modulated arc therapy (VMAT) with sequential or simultaneous integrated boost approaches to a total dose of 66–70 Gy with conventional or moderately accelerated fractionation (2–2.2 Gy per fraction). Radiotherapy procedures were previously reported [13,14,15]. 

Concomitant chemoradiation (c-CHT-RT) was based on IMRT plus cisplatin (CDDP) 100 mg/m^2^ every 3 weeks or cisplatin 50 mg/m^2^ delivered weekly. According to medical oncologist decision, patients who were deemed unfit for chemotherapy may not have received systemic treatment or could have changed cisplatin dosage or drug substitution, either a shift to 3-weekly carboplatin (CBDCA) or, for OPC only, cetuximab. In this case, cetuximab was administered at a loading dose of 400 mg/m^2^ before IMRT, followed by weekly maintenance doses of 250 mg/m^2^ until IMRT completion. OPC patients were treated with definitive IMRT with or without concomitant systemic therapy and induction chemotherapy according to disease stage, in agreement with international guidelines [16] and with institutional policies [17]. When delivered, induction chemotherapy (I-CHT) consisted of the TPF regimen: cisplatin 75 mg/m^2^ on day 1, docetaxel 75 mg/m^2^ on day 1 plus 5-fluorouracil 750 mg/m^2^/day, on days 1–4, every 3 weeks for two or three cycles. I-CHT was used until 2013, when a lack of benefit of its addition over upfront concurrent chemoradiation was observed [18,19,20].

In NPC patients, I-CHT with the TPF regimen was added in the case of diseases with a potential higher risk of distant metastasis (e.g., high burden N2 or N3 stage and/or T4 and/or elevated plasmatic EBV DNA at baseline), according to the EURACAN-ESMO guidelines [4]. 

After RT completion, patients were clinically evaluated at predefined intervals, every 3–6 months for the first 3 years and annually thereafter, as per international guidelines [4,21]. 

### 2.3. Statistical Analysis 

The distributions of demographic and clinicopathological variables were determined. 

To detect different distributions of the continuous variables in the different study populations, the Wilcoxon–Mann–Whitney exact test adjusted for tied values [22] or the Kruskal–Wallis rank sum test [23] were used, as appropriate. Similarly, the Fisher exact test or Fisher–Freeman–Halton test [24] were used to test categorical variables in the different study populations, as appropriate. Multiple testing was taken into account using the Benjamini and Hochberg procedure [25]. 

In the analysis of the association with other factors, age was included as a continuous variable. The sub-distributions of age in the study populations by patient and treatment characteristics were tested and adjusted using the same methods mentioned above for the continuous variables in the different study populations. 

The primary endpoints of this study were the assessments of differences in overall and disease-free survivals (OS and DFS, respectively) according to age in the three study cohorts (OPC HPV-positive; OPC HPV-negative; NPC EBER-positive). Survival times (OS and DFS) were computed starting from the date of diagnosis to death from any cause and recurrence of tumor or death from any cause, respectively. 

As a secondary endpoint, post-relapse OS was calculated on the patients’ subgroup with recurrence starting from the date of recurrence to death from any cause.

The association between the survival endpoints and the putative prognostic factors was studied using multivariable Cox models. The model results are shown in terms of the hazard ratio (HR), with 95% confidence interval (95% CI) and the Wald test *p*-value. Age was included in the multivariable models as a continuous variable using three-knots restricted cubic splines [26]. 

Several confounding factors could affect the proper investigation of the role of age on the survival endpoints. Therefore, we built a “dishomogeneity score” (used as the adjustment factor in all the multivariable models) as the linear predictor from a regression model in which age was the dependent variable and the covariates were the putative confounding factors (smoking status, stage, ACE-27 score, chemotherapy dose intensity—defined as the actual dose of chemotherapy delivered to a patient divided by the theoretical dose planned for the patient in the curative setting—and treatment strategy). 

The significance level was set at 0.05. All the statistical analyses were conducted using R version 4.1.2 [27].

## 3. Results

### 3.1. Patient Characteristics 

A total number of 324 patients were analyzed: 146 HPV + OPC, 63 HPV− OPC and 115 EBER + NPC. Patient and treatment characteristics are shown in Table 1. 

Overall, patients had a median age of 56 years (range 18–86), 74.4% were males and 41.4% never smoked. Subjects without comorbidities (ACE-27 = 0) represented 52.2%, while 48.0% had a lower extent of tumor (AJCC 8th T, T1-T2) and 55.3% showed lower lymph nodes involvement (AJCC 8th N, N0-N2b for OPC and N0-N1 for NPC). 

To detect a difference in the characteristics between the study populations, the Kruskal–Wallis test *p*-value or Fisher–Freeman–Halton exact test *p*-value were reported in Table 1, too. While no statistically significant evidence of different proportions of males and females was detected between the study populations (*p* = 0.244), as for AJCC 8th T stage (*p* = 0.434), other patient characteristics were significantly dissimilar. The current or previous smokers were the 96.8% of HPV− OPC sample, a proportion significantly different (*p* < 0.001) from the 65.8% of HPV+ OPC and the 27.0% of EBER+ NPC. The frequency of EBER+ NPC without comorbidities (ACE-27 equal to 0) was significantly higher (68.7%) compared to the one observed in the OPC populations (43.8% in HPV+ and 41.3% in HPV−, *p* < 0.001). A lower lymph nodes involvement was detected in 91.8% of HPV+ OPC patients, while the same frequency was decreased in HPV− OPC (55.6%) and, particularly, in EBER+ NPC (8.7%, *p* < 0.001).

By focusing on age across study cohorts, we observed that EBER+ NPC patients were significantly (*p* < 0.001) younger (median = 49 years) than patients in OPC cohorts (median for HPV+ was 59 years; median for HPV− 61 years). 

Table 2 shows age sub-distributions, described by using the median and first to third quartiles, in the overall sample and in the study cohorts by patient and treatment characteristics. The cohort-specific *p*-values were adjusted for multiple testing. 

Regarding tumor characteristics, age is similarly distributed within study cohorts for both AJCC 8th edition T (T1-T2 vs. T3-T4 WMW test *p*-values: HPV+ OPC 0.276; both HPV− OPC and EBER+ NPC 0.823) and N (low vs. high nodes invasion WMW test *p*-values equal to 0.556 in the three cohorts). 

In the same way, no evidence of different age distributions was detected by comparing males and females (WMW test *p*-values: HPV+ OPC 0.113; both HPV− OPC and EBER+ NPC 0.895) or high and low-grade toxicities (G1-2-3 vs. G3-4 WMW test *p*-values equal to 0.823 in the three cohorts).

Non-smoking HPV+ OPC subjects were significantly (*p* = 0.039) younger (median age = 55) than smokers (median age = 61). The median age of EBER+ NPC patients was equal to 49 years independent of smoking status, while the comparison was not possible for the HPV- OPC cohort because there were only two non-smokers. 

The presence of comorbidities was significantly (*p* = 0.001) attributed to older patients both in the HPV+ OPC and the EBER+ NPC cohorts: respectively, the median age was 61 and 58.5 years versus 56 and 44 years in patients without comorbidities. On the contrary, no statistically significant association (*p* = 0.402) was detected among HPV− OPC patients when comparing the age distributions of subjects with or without comorbidities. 

### 3.2. Treatments 

**Figure 1:**
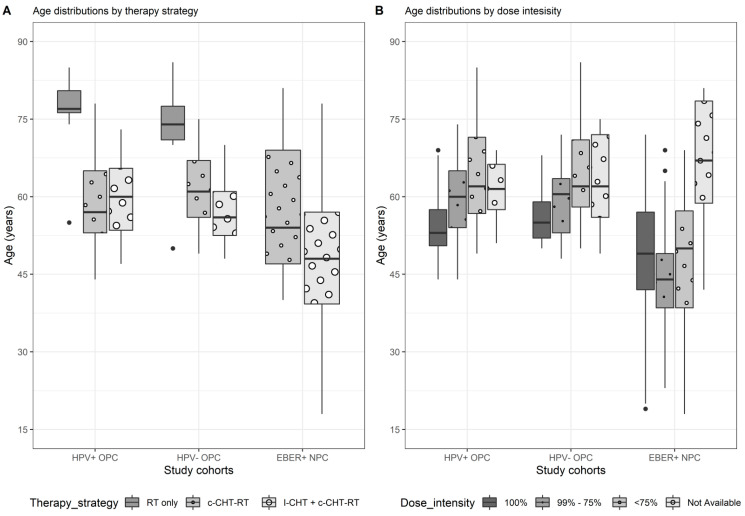
Age distribution by therapy strategy (**A**) and by categorized dose intensity (**B**). Abbreviation: Radiotherapy (RT); concurrent chemo-radiotherapy (RT+CT); induction chemotherapy followed by concomitant chemo-radiotherapy (RT+CT+IND).

The strongest differences in the age sub-distributions, independently of study cohorts, were found when comparing therapy strategies (*p*-values: HPV+ OPC, 0.001; HPV− OPC, 0.002; EBER+ NPC, 0.005). As reported in Table 2 and in Figure 1A, OPC patients treated with exclusive radiotherapy were systematically older (median age of 77 years in HPV+, *p* = 0.001; 74 years in HPV−, *p* = 0.002) than those treated with c-CHT-RT (57 years in HPV+ and 61 in HPV−) or those with the addition of I-CHT (60 years in HPV+ and 56 in HPV−). In the NPC cohort, which did not receive RT only, patients treated with I-CHT were significantly younger (median age of 48 years) than those who received c-CRT only (median age of 54 years, *p* = 0.005).

By shifting the focus onto associations between age and dose intensity, whose sub-distribution is depicted in Figure 1B and described in Table 2, it can be noticed that HPV+ OPC subjects receiving a full dose were significantly (*p* < 0.001) younger (median age = 53) compared to incomplete administration (median age in the 99–75% and <75% dose intensity class was 60 and 62 years, respectively). A similar behavior, despite not statistically significant (*p* = 0.091), can be observed on the HPV− OPC population, while no difference in the sub-distributions of age was detected in the EBER + NPC sample (*p* = 0.163). 

### 3.3. Disease-Free Survival and Overall Survival

In the study population, the median follow-up was 64.6 months (IQR 54.5–77.2). The five-year DFS was 85.3% in HPV+ OPC, 43.7% in HPV− OPC and 71.4% in NPC (*p* < 0.001). In these three groups, 5-year OS was 88.5%, 61.7% and 86.4% (*p* < 0.001), respectively (Supplementary Table S1).

The results of the adjusted multivariable Cox analysis for OS and DFS are shown in Table 3. Non-adjusted analysis is reported in Supplementary Table S2. As far as OS is concerned, after adjustment accounting for the confounding factors associated with age (see Section 2.3), the HR of HPV− vs. HPV+ OPC was 3.37 (95% CI 1.46–7.77) and the HR of NPC vs. HPV+ OPC 4.06 (95% CI 1.53–10.79) (*p* = 0.004). The HR of age 65 versus 50 years was 1.89 (95% CI 0.45–7.84) for HPV+ OPC, 0.91 (95% CI 0.29–2.89) for HPV− OPC and 1.99 (95% CI 0.9–4.39) for NPC (*p* = 0.395).

The HR for DFS was 5.37 (95% CI 2.64–10.93) in HPV− vs. HPV+ OPC patients, and 5.21 (95% CI 2.38–11.40) for NPC patients with respect to HPV+ OPC ones (*p* < 0.001). The HR of an age of 65 versus 50 years was 1.50 (95% CI 0.47–4.77) for HPV+ OPC, 0.67 (95% CI 0.26–1.73) for HPV− OPC and 1.13 (95% CI 0.67–1.91) for NPC (*p* = 0.852). 

In the 82 subjects with disease recurrence, the multivariable adjusted model showed that, for post-relapse OS, the HR between the elderly and young people was 2.53 (95% CI 0.40–16.00) in HPV+ OPC, 0.92 (95% CI 0.29–2.92) in HPV− OPC and 3.43 (95% CI 1.49–7.89) in NPC. 

## 4. Discussion

This retrospective study primarily analyzed how the interplay between age, virus status, and treatment approach impacted on outcomes in NPC and OPC patients.

In the original non-adjusted model, a statistically significant higher risk of death was observed in the elderly population, while it was borderline significant for DFS in the same direction. 

After adjusting for stage, smoking status, comorbidities, treatment strategy, and intensity, no statistical differences were observed in young subjects compared to old subjects, especially in virus-related cancers.

On this basis, we confirm the evidence found in literature that the factors used to adjust the models play a relevant prognostic role with a differential impact on virus vs. non virus-related tumors. 

As expected, in the present study, the frequency of smokers and subjects with more comorbidities were higher in the global elderly population [28,29,30]. In line with data from the literature, these factors may justify higher competing mortality such as non-cancer causes like comorbidities and/or second primary malignancies [31,32,33]. 

Consistently with clinical best practices, older patients were more likely to receive less aggressive treatments with less concomitant and induction chemotherapy [4]. Also, we showed that older patients received an inferior dose intensity of concurrent chemotherapy than younger ones. Even with physician’s selection of the treatment strategy, the compliance of the patients with chemotherapy is reduced with increasing age, thus underlining the need for more accurate evaluations of treatment strategies in elderly subjects [34].

In addition, we showed that, after adjusting for a linear predictor of age, NPC had worse outcomes (both DFS and OS) than HPV+ OPC, while the latter group confirmed its well-known [35,36] better prognosis when compared with its HPV− counterpart. 

In this context, in our study population HPV− OPC patients were slightly older and more frequently smokers than what was observed in the HPV+ OPC patient cohort. This is in line with the available literature in the field [36,37].

Although both are virally related cancers, NPC and HPV+ OPC have different biology and natural history that may justify these observations [5,38]. Nonetheless, we observed that the statistically negative effect of age in virus-related cancers was smoothened after model adjustment. Interestingly, this effect was not seen in the HPV− counterpart suggesting a different interplay between age, treatment, tumor and host characteristics in the two groups. 

With the caveat of the limited number of HPV− OPC patients considered in this study and given the specific type of the aforementioned factors, we speculate that the intrinsic characteristics of the non-virus related tumors might overcome them. By contrast, in virus-related cancers, an approach acting on modifiable factors such as dose intensity and treatment strategy is justified independently of age. 

According to our results, age in itself should not be considered as an independent prognostic factor. Indeed, in the literature, the prognostic role of age in HNC is controversial [39,40,41,42,43,44]. In particular, age has been found to play a role for the prognosis of OPC with a cut-off point of 65 years [37,45,46]. Similarly to OPC, for NPC patients age significantly impacts on survival [47,48].

In our study, older age correlated with a worse post-relapse OS in NPC patients, but not in OPC ones. This might reflect the opportunity of a less aggressive salvage treatment for elderly patients [49,50]. 

The main limitations of our analysis are its retrospective nature and the small number of patients over 65 years. Furthermore, the fact that systemic treatments were chosen at the discretion of the treating oncologist created inherently disparate subgroups for each regimen, making direct comparisons harder to be interpreted. 

Given the heterogeneity of the older population, it is crucial to develop scientifically validated tools for assessing the fitness of an older patient to receive multimodal treatment beyond simply age and performance status [51]. 

In this regard, standard geriatric assessment tools, such as the comprehensive geriatric assessment may be helpful [52] and in accordance with our observations especially in virus-related tumor patients where an accurate host profiling might lead to a more appropriate treatment approach.

In one of the largest geriatric chemoradiation studies to date, a Karnofsky performance status ≤80, Charlson index ≥3 and weekly platinum were associated with lower treatment completion rates for patients undergoing chemoradiation [53]. Similarly, ACE-27 was shown to be a predictor of outcome in a retrospective cohort of NPC patients, thus underlining the necessity to comprehensively assess elderly patients [54].

## 5. Conclusions

Given the results, we may posit that age in itself should be considered as the expression of an array of host and tumor-related features rather than an independent prognostic factor.

## Figures and Tables

**Table 1:** Patient characteristics overall and according to study cohorts.

Variable	Statistic/Levels	Overall (*n* = 324)	HPV+ OPC (*n* = 146)	HPV− OPC (*n* = 63)	EBER+ NPC (*n* = 115)	*p*-Value
Age (years)	Mean (SD)	56.2 (11.9)	59.7 (8.5)	61.3 (8.5)	48.8 (13.6)	
	Median (IQR)	56.0 (50.0–64.0)	59.0 (53.0–66.0)	61.0 (54.5–67.5)	49.0 (40.0–58.0)	<0.001
	Min–Max	18–86	44–85	48–86	18–81	
Gender	Females (*n*,%)	83 (25.6)	36 (24.7)	12 (19.0)	35 (30.4)	0.314
	Males (*n*,%)	241 (74.4)	110 (75.3)	51 (81.0)	80 (69.6)	
Smoker	Yes (*n*,%)	188 (58.0)	96 (65.8)	61 (96.8)	31 (27.0)	
	No (*n*,%)	134 (41.4)	48 (32.9)	2 (3.2)	84 (73.0)	<0.001
	Missing	2 (0.6)	2 (1.3)	0 (0.0)	0 (0.0)	
ACE 27	0 (*n*,%)	169 (52.2)	64 (43.8)	26 (41.3)	79 (68.7)	
	1 (*n*,%)	112 (34.6)	64 (43.8)	26 (41.3)	22 (19.1)	<0.001
	2 (*n*,%)	36 (11.1)	16 (11.0)	10 (15.9)	10 (8.7)	
	3 (*n*,%)	7 (2.1)	2 (1.4)	1 (1.5)	4 (3.5)	
AJCC 8th T	T1–T2 (*n*,%)	155 (48.0)	70 (48.3)	26 (41.3)	59 (51.3)	0.434
	T3–T4 (*n*,%)	168 (52.0)	75 (51.7)	37 (58.7)	56 (48.7)	
AJCC 8th N	N0–N2b (*n*,%)	169 (52.2)	134 (91.8)	35 (55.6)	-	<0.001
	N2c–N3 (*n*,%)	40 (12.3)	12 (8.2)	28 (44.4)	-	
	N0–N1 (*n*,%)	10 (3.1)	-	-	10 (8.7)	-
	N2–N3 (*n*,%)	105 (32.4)	-	-	105 (91.3)	
Therapy strategy	Exclusive RT (*n*,%)	15 (4.6)	8 (5.5)	7 (11.1)	0 (0.0)	
	RT + CT (*n*,%)	161 (49.7)	107 (73.3)	33 (52.4)	21 (18.3)	<0.001
	RT + CT + IND (n,%)	148 (45.7)	31 (21.2)	23 (36.5)	94 (81.7)	
Toxicity	G3-4 (*n*,%)	179 (55.2)	70 (47.9)	21 (33.3)	88 (76.5)	<0.001
	G0-1-2 (*n*,%)	145 (44.8)	76 (52.1)	42 (66.7)	27 (23.5)	
Dose Intensity (%)	Mean (SD)	77.5 (23.5)	78.4 (23.9)	71.3 (28.5)	79.6 (19.6)	
	Median (IQR)	83 (67–100)	83 (67–100)	83 (67–92)	83 (66–100)	0.367
	Min–Max	0–100	0–100	0–100	23–100	
	Not available (*n*,%)	33 (10.2)	16 (11.0)	9 (14.3)	8 (7.0)	

Kruskal–Wallis test *p*-value or Fisher–Freeman–Halton Exact test, as appropriate, adjusted for multiple testing using Benjamini and Hochberg’s correction. Information not available for one patient. Abbreviations: oropharyngeal and nasopharyngeal cancers (OPC and NPC, respectively); human papilloma virus (HPV); Epstein–Barr virus–encoded small RNA (EBER); adult comorbidity evaluation 27 (ACE-27); radiotherapy (RT); concurrent chemo-radiotherapy (RT + CT); induction chemotherapy followed by concomitant chemo-radiotherapy (RT + CT + IND); standard deviation (SD); interquartile range (IQR).

**Table 2:** Median age and interquartile range in the overall sample and in the study cohorts by patients’ and treatment characteristics.

		Median Age (IQR) and *p*-Value ^a^ by Variables’ Levels	Median Age (IQR) and *p*-Value ^b^ by Variables’ Levels and by Study Cohort		
Variable	Levels	Overall (*n* = 324)	HPV+ OPC (*n* = 146)	HPV− OPC (*n* = 63)	EBER+ NPC (*n* = 115)
Gender	Females	55.0 (47.0–62.0)	56.0 (52.0–61.0)	61.5 (55.5–65.2)	47.0 (41.0–60.0)
	Males	57.0 (50.0–65.0)	60.0 (54.0–66.8)	61.0 (54.5–67.5)	49.5 (40.0–57.2)
		0.040	0.113	0.895	0.895
Smoker	Yes	60.0 (52.0–66.0)	61.0 (54.8–66.2)	61.0 (55.0–68.0)	49.0 (40.0–58.0)
	No	52.0 (44.0–59.8)	55.0 (52.0–61.0)	-	49.0 (40.0–58.0)
		<0.001	0.039	-	0.975
ACE 27	0	61.0 (54.0–68.0)	56.0 (51.0–61.0)	59.5 (54.2–63.5)	44.0 (37.5–52.0)
	≥1	52.0 (45.0–60.0)	61.5 (55.0–67.0)	61.0 (55.0–70.0)	58.5 (49.0–65.5)
		< 0.001	0.001	0.402	<0.001
AJCC 8th T	T1–T2	55.0 (49.0–64.0)	57.5 (52.0–65.0)	61.5 (54.0–69.8)	49.0 (40.0–57.0)
	T3–T4	57.5 (50.0–64.2)	60.0 (54.5–66.5)	61.0 (55.0–66.0)	49.0 (39.2–58.2)
		0.220	0.276	0.823	0.823
AJCC 8th N ^c^	Low nodes invasion	52.0 (44.0–62.0)	58.5 (53.0–65.8)	61.0 (55.0–62.5)	42.5 (40.0–55.8)
	High nodes invasion	59.0 (53.0–65.0)	60.5 (57.0–66.2)	61.0 (53.8–69.5)	49.0 (40.0–58.0)
		<0.001	0.556	0.556	0.556
Therapy strategy	RT only	77.0 (73.0–79.5)	77.0 (76.2–80.5)	74.0 (71.0–77.5)	-
	RT + CT	59.0 (53.0–66.0)	57.0 (53.0–65.0)	61.0 (56.0–67.0)	54.0 (47.0–69.0)
	RT + CT + IND	52.0 (44.0–60.0)	60.0 (53.5–65.5)	56.0 (52.5–61.0)	48.0 (39.2–57.0)
		<0.001	0.001	0.002	0.005
Toxicity	G3-4	55.0 (48.0–64.5)	59.0 (53.0–66.8)	61.0 (55.0–68.0)	49.0 (40.0–58.2)
	G0-1-2	58.0 (52.0–64.0)	59.0 (53.8–65.0)	61.0 (54.2–66.8)	49.0 (40.0–56.0)
		0.038	0.823	0.823	0.823
Dose intensity (%)	100	53.0 (48.0–58.0)	53.0 (50.5–57.5)	55.0 (52.0–59.0)	49.0 (42.0–57.0)
	99–75	56.0 (48.0–62.5)	60.0 (54.0–65.0)	60.5 (53.0–63.5)	44.0 (38.5–49.0)
	<75	59.0 (50.0–67.0)	62.0 (56.8–71.5)	62.0 (58.0–71.0)	50.0 (38.5–57.2)
		0.004	<0.001	0.091	0.163
	Not available	62.0 (58.0–69.0)	61.5 (57.5–66.2)	62.0 (56.0–72.0)	67.0 (58.8–78.5)

^a^: Wilcoxon–Mann–Whitney exact test *p*-value or Kruskal–Wallis rank sum test *p*-value, as appropriate; ^b^: Wilcoxon–Mann–Whitney exact test *p*-value or Kruskal–Wallis rank sum test *p*-value, as appropriate, adjusted for multiple testing by applying Benjamini-Hochberg procedure; ^c^: low nodes invasion: for OPC, N0-N2b; for NPC, N0-N1. High nodes invasion: for OPC, N2c-N3; for NPC, N2-N3.

**Table 3:** Multivariable analyses of overall survival (OS) and disease-free survival (DFS).

			OS		DFS	
Covariates	Reference	Comparison	HR (95% CI)	*p*-Value	HR (95% CI)	*p*-Value
Age (years)	OPC+	65 vs. 50	1.89 (0.45–7.84)	0.395	1.50 (0.47–4.77)	0.852
	OPC−	65 vs. 50	0.91 (0.29–2.89)		0.67 (0.26–1.73)	
	NPC	65 vs. 50	1.99 (0.90–4.39)		1.13 (0.67–1.91)	
Study cohort	Age 56 years	OPC− vs. OPC+	3.37 (1.46–7.77)	0.004	5.37 (2.64–10.93)	<0.001
	Age 56 years	NPC vs. OPC+	4.06 (1.53–10.79)		5.21 (2.38–11.40)	
Interaction term				0.751		0.749

The age values (i.e., 50, 56, and 65) are, respectively, the 1st quartile, the median and the cut-off used for defining the age classes young (<65 years) and old (≥65 years). Together with age and cohort, the models also included their interaction to investigate the differential effect of age in the three cohorts and, symmetrically, the effect of cohorts at varying ages. The first is represented in the upper panel of the table (“Age (years)”), while for the second we only estimated the effect of the cohort at median age (lower panel of the table “Study cohort”). The models also included a score to adjust for confounding factors possibly affecting the role of age on the three endpoints (see the Section 2.3). The *p*-value refers to the 2-sided Wald test. Abbreviations: oropharyngeal and nasopharyngeal cancers (OPC and NPC, respectively); human papilloma virus (HPV) status (positive or negative, + or −); hazard ratio (HR); 95% confidence interval (95% CI); Overall Survival (OS); Disease-Free Survival (DFS).

## Data Availability

The datasets analyzed for this study may be available upon reasonable request to the Authors.

## Supplementary Materials

The following supporting information can be downloaded at: https://www.mdpi.com/article/10.3390/cancers14246170/s1, Table S1: Survival endpoints estimates; Table S2: Non adjusted multivariable analyses of overall survival (OS) and disease-free survival (DFS).
