# Supplementary material for: The Interplay between Age and Viral Status in EBV-Related Nasopharyngeal and HPV-Related Oropharyngeal Carcinoma Patients
Source: Cancers (Basel). 2022 Dec 14;14(24):6170. doi: 10.3390/cancers14246170 (PMC9777049; doi:10.3390/cancers14246170)
Supplement: Supplementary file 1 [file cancers-14-06170-s001.zip › cancers-2071975-supplementary.pdf]

Supplementary Material

Table S1. Survival endpoints estimates.

	Overall (*n* = 324)		HPV+ OPC (*n* = 146)		HPV- OPC (*n* = 63)		EBER+ NPC (*n* = 115)	
Median follow-up time (IQR) (months)	64.6	(54.5 - 77.2)	64.4	(54.5 - 80.0)	66.1	(51.9 - 84.4)	64.3	(56.0 - 70.2)
2-year OS (95% CI) (%)	91.2	(88.2 - 94.4)	93.8	(90.0 - 97.8)	79.0	(69.5 - 89.8)	92.9	(88.2 - 97.8)
2-year DFS (95% CI) (%)	81.3	(77.2 - 85.7)	91.1	(86.5 - 95.8)	57.9	(46.8 - 71.7)	78.9	(71.7 - 86.8)
5-year OS (95% CI) (%)	82.7	(78.5 - 87.1)	88.5	(83.4 - 94.0)	61.7	(50.1 - 76.1)	86.4	(80.2 - 93.0)
5-year DFS (95% CI) (%)	72.8	(68.0 - 77.9)	85.3	(79.7 - 91.3)	43.7	(32.7 - 58.5)	71.4	(63.4 - 80.3)

Abbreviations: oropharyngeal and nasopharyngeal cancers (OPC and NPC, respectively); Human PapillomaVirus (HPV); Epstein-Barr virus–encoded small RNA (EBER); overall survival (OS); disease-free survival (DFS); interquartile range (IQR); 95% confidence interval (95% CI).

Table S2. Non adjusted multivariable analyses of overall survival (OS) and disease-free survival (DFS).

Covariates	Reference	Comparison	OS		DFS	
**Age (years)**	**OPC+**	**65 vs 50**	**HR (95% CI)**	**p-value**	**HR (95% CI)**	**p-value**
	OPC-	65 vs 50	2.47 (0.66-9.25)		1.98 (0.67-5.86)	
	NPC	65 vs 50	1.12 (0.35-3.58)	0.004	0.82 (0.31-2.12)	0.058
Study cohort	Age 56 years	OPC- vs OPC+	2.63 (1.22-5.67)		1.51 (0.92-2.47)	
	Age 56 years	NPC vs OPC+	3.60 (1.55-8.33)	0.005	5.45 (2.66-11.17)	<0.001
Interaction term			2.60 (1.02-6.61)		3.30 (1.57-6.93)	
				0.561		0.628

The age values (i.e. 50, 56, and 65) are, respectively, the 1st quartile, the median and the cut-off used for defining the age classes young (<65 years) and old (≥65 years). Together with age and cohort, the models included also their interaction to investigate the differential effect of age in the three cohorts and, symmetrically, the effect of cohort at varying age. The first is represented in the upper panel of the table ("Age (years)"), while as for the second we only estimated the effect of cohort at median age (lower panel of the table "Study cohort"). p-value refers to the 2-sided Wald test. Abbreviations: oropharyngeal and nasopharyngeal cancers (OPC and NPC, respectively); Human PapillomaVirus (HPV) status (positive or negative, + or -); hazard ratio (HR); odds ratio (OR); 95% confidence interval (95% CI).
